# Workflow and Atlas System for Brain-Wide Mapping of Axonal Connectivity in Rat

**DOI:** 10.1371/journal.pone.0022669

**Published:** 2011-08-01

**Authors:** Izabela M. Zakiewicz, Yvette C. van Dongen, Trygve B. Leergaard, Jan G. Bjaalie

**Affiliations:** Centre for Molecular Biology and Neuroscience, Institute of Basic Medical Sciences, University of Oslo, Oslo, Norway; University of Queensland, Australia

## Abstract

Detailed knowledge about the anatomical organization of axonal connections is important for understanding normal functions of brain systems and disease-related dysfunctions. Such connectivity data are typically generated in neuroanatomical tract-tracing experiments in which specific axonal connections are visualized in histological sections. Since journal publications typically only accommodate restricted data descriptions and example images, literature search is a cumbersome way to retrieve overviews of brain connectivity. To explore more efficient ways of mapping, analyzing, and sharing detailed axonal connectivity data from the rodent brain, we have implemented a workflow for data production and developed an atlas system tailored for online presentation of axonal tracing data. The system is available online through the Rodent Brain WorkBench (www.rbwb.org; Whole Brain Connectivity Atlas) and holds experimental metadata and high-resolution images of histological sections from experiments in which axonal tracers were injected in the primary somatosensory cortex. We here present the workflow and the data system, and exemplify how the online image repository can be used to map different aspects of the brain-wide connectivity of the rat primary somatosensory cortex, including not only presence of connections but also morphology, densities, and spatial organization. The accuracy of the approach is validated by comparing results generated with our system with findings reported in previous publications. The present study is a contribution to a systematic mapping of rodent brain connections and represents a starting point for further large-scale mapping efforts.

## Introduction

The vast numbers of neurons in the brain are connected with a hugely larger number of synapses. The axonal pathways they form make up the wiring of the brain, an important basis for understanding of brain functions under normal as well as pathological conditions [1]–[4]. Axonal pathways can be studied at various levels of granularity, ranging from mapping of major fiber pathways (trajectories) using macroscopic dissection or novel magnetic resonance imaging (MRI) based tractography techniques [5]–[9], to detailed reconstruction of individual axons and synaptic contacts using confocal or electron microscopy [10]–[14]. An intermediate level of analysis is delivered by use of axonal tract-tracing methods, which is suitable for mapping the specific axonal connections of limited populations of neurons [15]–[19], see also [20]). Axonal tracing studies yield highly specific, detailed visualizations of axonal connections in histological sections. Systematic efforts to create connectivity databases building on published axonal tracing data [21]–[25] have proven helpful for combining key information from a multitude of experimental investigations. However, the currently available systems primarily capture the presence of connections. Only limited information is provided on morphology, densities, and spatial organization. Moreover, since original data are typically not available through the conventional publication formats, or through the current database systems, more detailed data analysis and re-investigation of the data cannot be performed.

In the context of determining the wiring patterns of the brain, including detailed anatomical organization of individual connections, we have initiated a series of axonal tracing experiments together with a systematic approach for collecting, managing, analyzing, and disseminating the histology-based data generated by the experiments. To overcome in particular the current limitations of axonal tracing studies, as outlined above, we have aimed at 1) delivering a brain-wide mapping of connections in each experiments, 2) capturing multiple aspects of the anatomical organization of the connections, and 3) facilitating comparison across investigations by providing detailed metadata and reference to standardized atlas diagrams. More specifically, we present 1) a comprehensive collection of brain-wide histological image data from six tract-tracing experiments, in which axonal tracers were placed in the primary somatosensory cortex (SI), 2) a data system for management of these image data and associated metadata, including tools for online viewing and analysis of the image data, and 3) a workflow for systematic collection of tract-tracing data. We exemplify the use of the image collection and data system with an analysis of several efferent projections from the rat primary somatosensory cortex.

## Results

**Figure 1:**
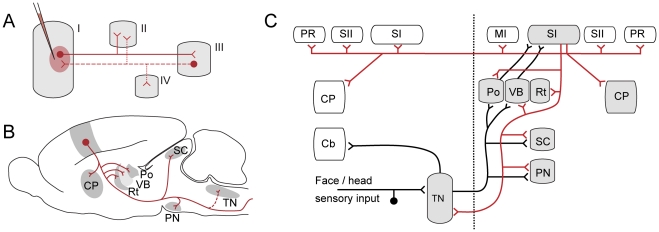
Diagrams of axonal tracing paradigm and connections of rat brain somatosensory cortex. (A) Schematic diagram showing the principles of anterograde axonal tracing. Following injection of an anterograde axonal tracer to a brain region (I), the tracer is taken up by neurons (red dot in region I) within the injection site, and anterogradely transported along efferent axons, yielding distinctly labeled axons and terminal fields (solid lines) in regions (II, III) receiving axonal projections from the injected region. While the tracer *Phaseolus vulgaris* leucoagglutinin only gives anterograde labeling (solid red lines), the bidirectional tracer biotinylated dextran amine, also gives rise to retrograde labeling of neurons (red dot in region II), as well as secondary anterograde labeling of collateral axons (dashed red lines to regions II and IV). (B) Diagram of the rat brain showing axonal projections from the primary somatosensory cortex to well-known ipsilateral (solid red lines) and contralateral (dashed red lines) subcortical targets. (C) Schematic wiring diagram (modified from [31]) showing the input and output relationships of the primary somatosensory cortex (SI). SI input from the trigeminal system is shown (solid black lines). SI output connections (solid red lines) reach several cortical and subcortical regions. Grey shading indicates regions investigated in the present study (Fig. 5). CP, caudate putamen; Cb, cerebellum; MI, primary motor cortex; PN, pontine nuclei; Po, posterior complex thalamus, PR, perirhinal cortex; Rt, reticular nucleus thalamus; SI, primary somatosensory cortex; SII, secondary somatosensory cortex; SC, superior colliculus; TN, trigeminal nuclei; VB, ventrobasal complex thalamus.

We have used axonal tracing techniques (Fig. 1) to map the connections of populations of neurons throughout the brain. Axonal tracer injections were placed within the cerebrocortical grey matter, restricted to body surface representations of the forelimb or whiskers in SI. The tracer injections gave rise to distinctly labelled axonal trajectories and terminal fields, seen as plexuses with profusely branching axons.

**Figure 2:**
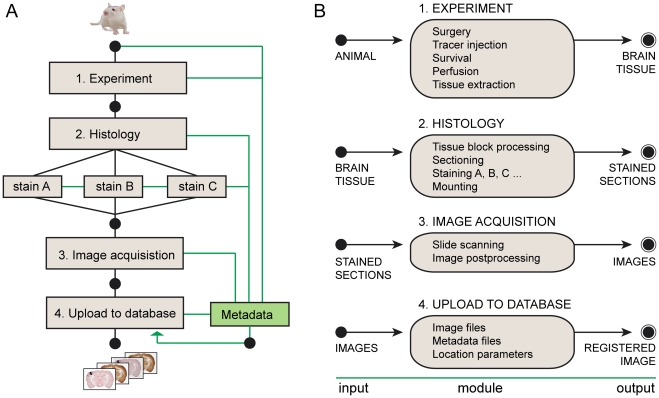
Workflow. (A) Flowchart showing four processing steps, beginning with an animal submitted to an axonal tract-tracing experiment, followed by tissue and image processing steps, to an end result consisting of section images in a database. For each step, information about experimental parameters and procedures (metadata) are stored together with the section images. (B) Diagram showing module details and the input and output elements of each module in the workflow.

**Figure 3:**
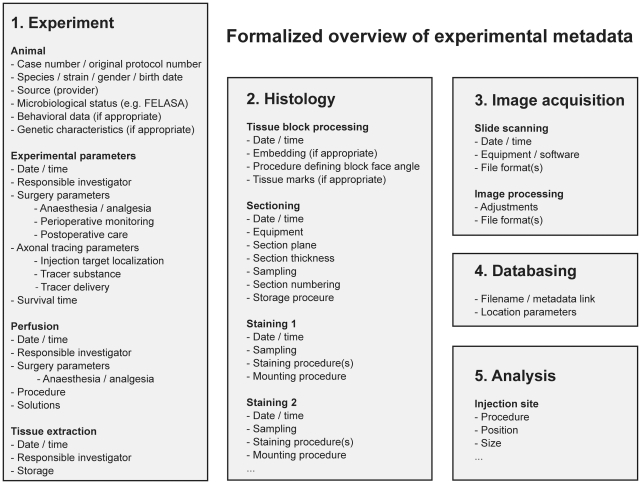
Formalized overview of experimental metadata recorded for axonal tracing experiments. Information deemed necessary for the interpretation and re-use of axonal tracing experiments, sorted according to the processing steps shown in Figure 2.

**Figure 4:**
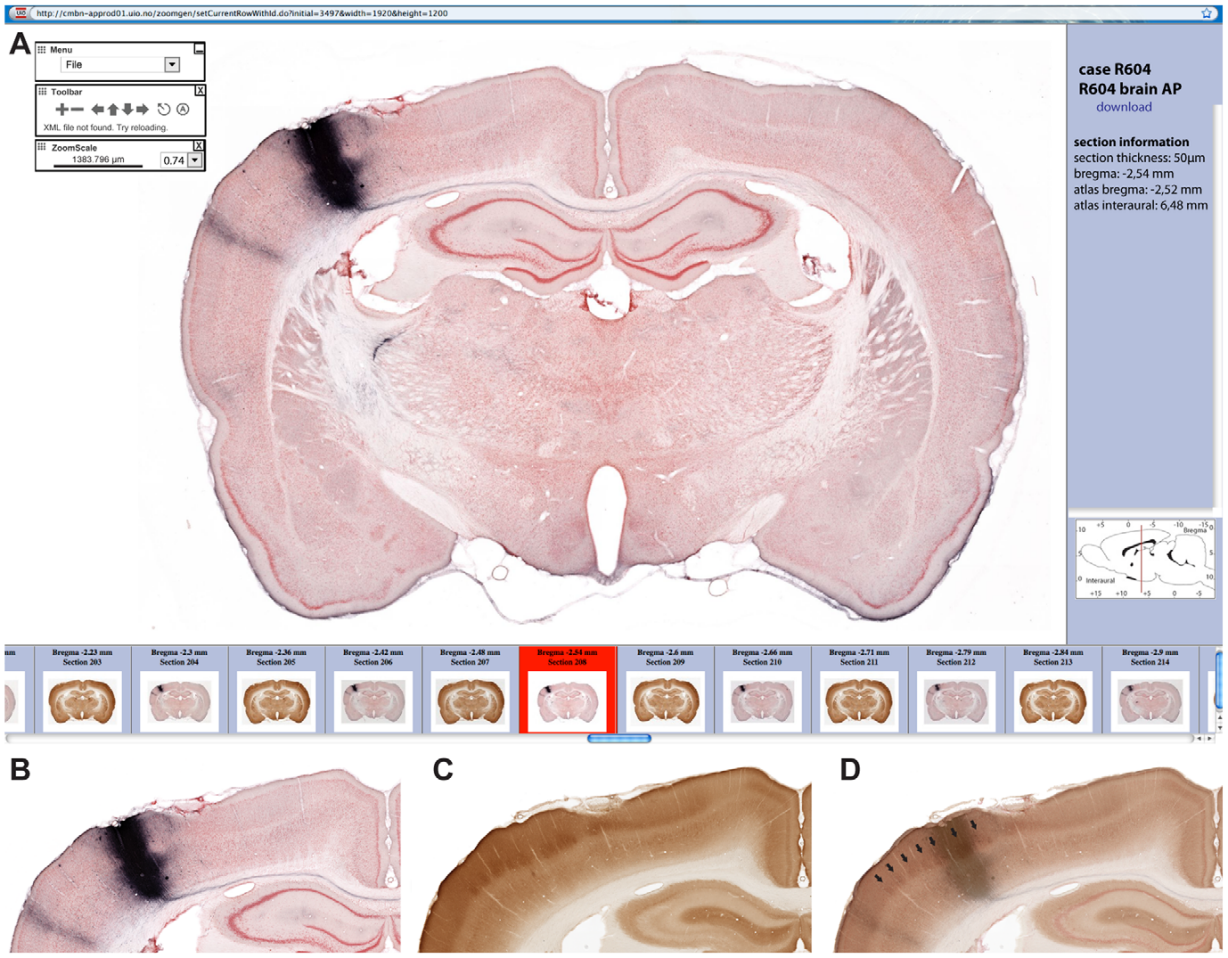
Graphical user interface of the online image repository. (A) Viewer tool providing access to a series of images organized by bregma levels. Thumbnail images (bottom row) provide overview of the available images, and selected images can be explored in the viewer panel by interactive zooming and panning. Metadata are available via a link in the viewer. (B–D) Evaluation of injection site location. Comparison of a BDA labeled section through the injection site (B) and a neighboring section (C) showing the SI barrel architecture (cytochrome oxidase staining). (D) Overlay of (B) and (C). Arrows indicate individual cytochrome oxidase positive SI whisker barrels.

**Figure 5:**
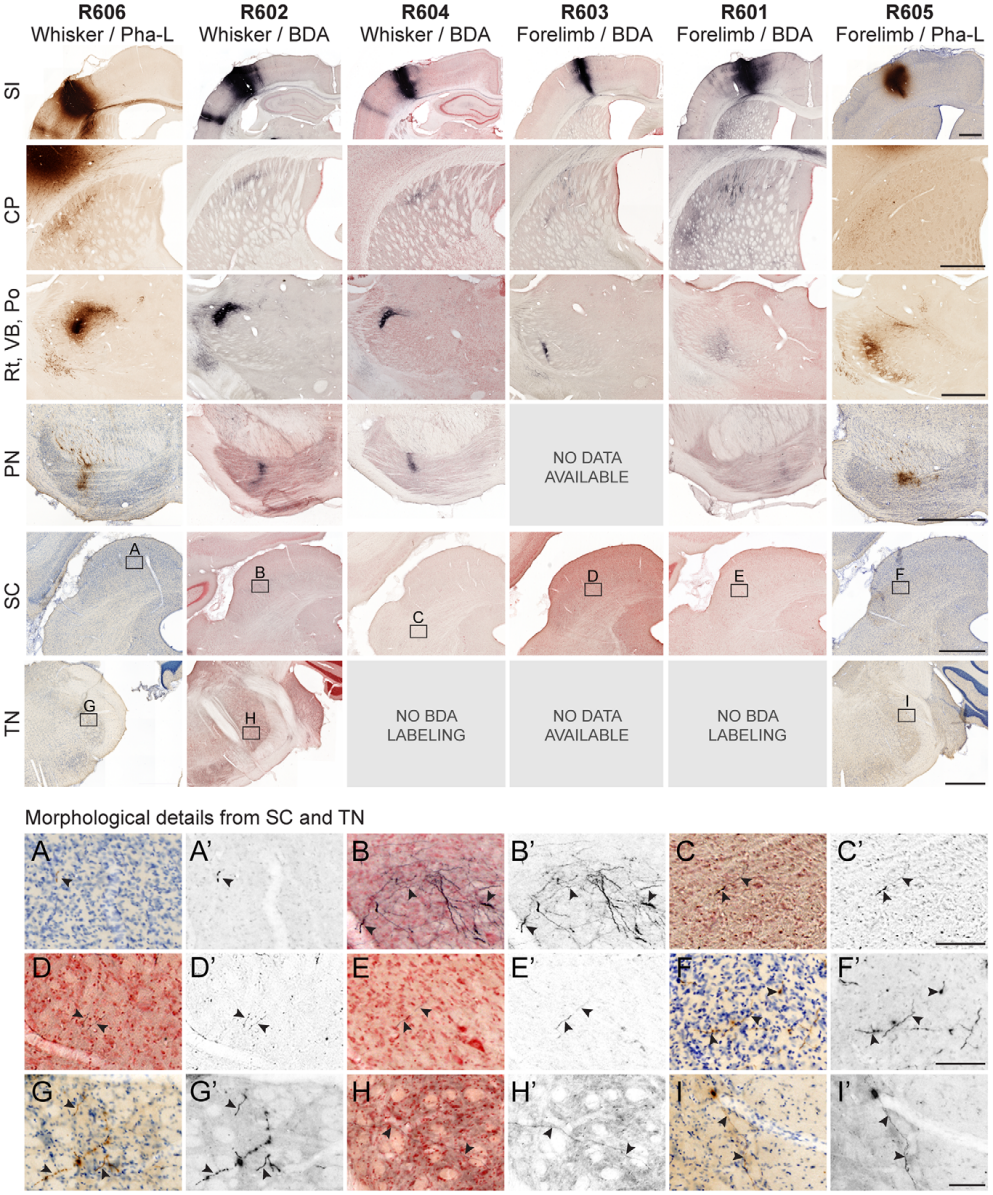
Overview of subcortical projections from SI whisker and forelimb representations. Analysis of subcortical projections in six experiments with tracer injections in SI whisker or forelimb representations (row 1). Images are sorted according to animals (columns) and location (rows). Comparison within rows 2–6 demonstrates that projections from SI whisker and SI forelimb representations, respectively, have similar axonal distributions. Comparison within columns demonstrates considerable variability in the amount of labeled axons present in the different regions receiving SI projections. The bottom panels (A–I) show morphological details from regions indicated by frames in the superior colliculus (row 5) and trigeminal nuclei (row 6). Panels A′–I′ show processed images with background staining removed to facilitate visualization of labeled fibers. CP, caudate putamen; PN, pontine nuclei; Po, posterior complex thalamus, Rt, reticular nucleus thalamus; SI, primary somatosensory cortex; SC, superior colliculus; TN, trigeminal nuclei; VB, ventrobasal complex thalamus. Scale bars, 1 mm (rows 1–6) and 100 µm (A–I).

**Figure 6:**
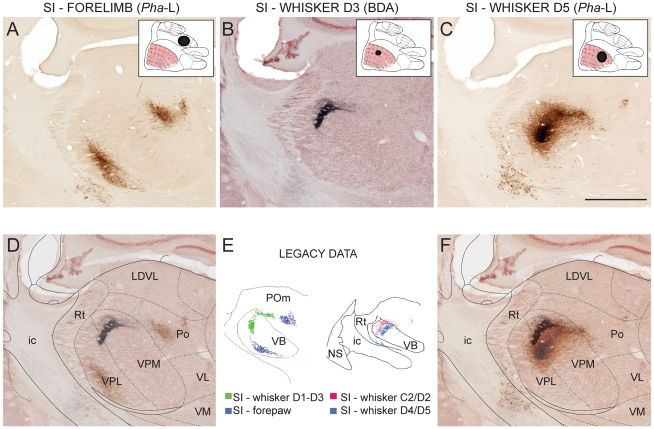
Topography of corticothalamic projections from different body part representations in primary somatosensory cortex (SI). (A–C) Images from corresponding locations in the thalamus from three experiments, showing labeled corticothalamic axonal plexuses. Inset figures indicate the localization and extent of injection sites. (D, F) Semitransparent overlays of image panels A and B, and B and C, showing distinct topographical distribution patterns in full agreement with earlier results from dual-tracing experiments. (E) Computerized plots showing topographical distribution of corticothalamic projections following SI tracer injections, modified from [51] and [50]. Scale bar: 1 mm.

To enhance the efficiency of collecting, managing, analyzing and disseminating these histological data, we employed a workflow with a series of modules, beginning with the animal experiment and ending with uploading of high resolution microscope images to a database and the registration of the images to a spatial framework (Fig. 2). All experimental data, consisting of section images and metadata for the experiment, are presented in the data system (Figs. 2A, 3). The histological sections are sampled at regular intervals (100 or 200 µm) across the entire brain, and the section images are captured with a section scanning technique providing a resolution at the level of individual fibers and cells. Series of images from each experiment are available through an online open access application, referred to as a digital atlas system. The system consists of an image repository accompanied by a viewing tool with interactive zooming and panning (Fig. 4). A panel containing a row of thumbnail images provides an overview of the complete series of section images and the assigned position of the sections in relation to standard atlas diagrams. The system is available via the Rodent Brain WorkBench portal (www.rbwb.org, Whole Brain Connectivity Atlas) and currently holds ∼1200 high-resolution section images from six axonal tract-tracing experiments, in which the anterograde tracers biotinylated dextran amine (BDA) or *Phaseolus vulgaris* leucoagglutin (*Pha*-L) were injected in specific SI body representations (Fig. 1). Using the data system, we demonstrate three possible routes for analyzing brain-wide connectivity at the level of regions, areas and nuclei, resulting in 1) mapping of the presence of connections (Fig. 5), 2) analysis of densities of labeling (Fig. 5), and 3) analysis of detailed topographical organization (Fig. 6).

We provide a brief description of the workflow and the management of data and metadata, before exemplifying the use of the system.

### Workflow for standardized brain-wide mapping of connections

The workflow begins with an animal, which is subject to an axonal tract-tracing experiment (Figs. 1 and 2). The result of this first step is a fixed brain containing a stereotaxically positioned tracer injection. In the context of such tracing experiments, the position and size of the injection site is a primary variable.

In our experiments, with the objective to trace the output pathways of a defined cortical region, the tracer was injected in the cortex at multiple locations along the needle tract, producing a columnar injection site involving all cortical layers, but not the underlying white matter (Figs. 4A–B, 5, top row). The tracer injections were targeted to specific SI body (whisker and forelimb) representations with use of stereotaxic coordinates measured as anterioposterior and mediolateral distances from bregma. The achieved positions were later confirmed by histological analysis of anatomical landmarks, including cytochrome oxidase staining (Fig. 4C–D; see below). Following the animal experiment and perfusion, the second step was the preparation of blocks of brain tissue, with use of standardized procedures for determining the block surface orientation and thereby the angle of sectioning [26]. We have chosen to employ a coronal section plane based on the flat skull position employed in commonly used rat brain atlas diagrams [27], [28]. This was achieved by use of external brain landmarks before dividing the brain in two parts with a single cut placed at the level of the mesencephalon. The brain blocks were marked unilaterally to ensure that sections were correctly oriented on the slides. In the third step, brain blocks were cut on a freezing microtome and free-floating sections were collected in individual wells. The number of sections lost at the beginning and end of each tissue block was estimated before consecutive serial numbers were assigned to all sections (including incomplete or lost sections). These numbers were later used for assigning positional coordinates to section images.

Following visualization of axonal labeling (immunohistochemistry on free-floating sections) in a regularly spaced series, a counterstaining was applied to reveal cytoarchitectonic features useful for determining the location of axonal labeling (Fig. 4A–B). In addition, alternate sections surrounding the injection site were stained for the enzyme cytochrome oxidase to visualize the characteristic SI-barrel architecture (Fig. 4C–D; [29]). Careful positioning of sections on the slides facilitated the image acquisition procedure and thereby minimized the need for image post-processing. Mosaic images were captured through a 10× objective using a semi-automatic robotic microscope system. Image post-processing steps were limited to rotation of images and necessary reformatting before exporting to the viewer application. Batches of images and metadata were uploaded to an administrator version of the data system, and anchored to corresponding rat brain atlas diagrams on the basis of anatomical landmarks and serial numbers of the sections. In this way, an anteroposterior level, indicated as distance from bregma [28], was assigned to each section image.

The endpoint of the workflow was thus a brain-wide series of high-resolution section images registered to an atlas, and made accessible via the Internet with use of an interactive viewer tool (Fig. 4A).

### Data and metadata

Each step in the workflow has input data, a set of procedures describing experiments or data analysis steps, and output data (Fig. 2). The different procedure descriptions are collected together with information about the animal, materials, and different experimental parameters. (Figs. 2A, 3). In line with recent guidelines proposed for metadata from electrophysiological experiments [30], we present a set of metadata with information elements essential for interpretation and reuse of axonal tract-tracing data (Fig. 3). Relative to animals, the metadata include biographical information and parameters related to the tract-tracing experiments, including documentation of the tracer substance and characteristics of the injection sites. Relative to tissue samples, the metadata include histological procedures, block face angle, tissue marks to identify orientation, section sampling, and staining. Relative to the acquired image data, the metadata include equipment and procedures for collecting the data, as well as data formats. The collected metadata are made available via the online data system.

### Example analyses

The Whole Brain Connectivity Atlas provides access to comprehensive series of experimental image data containing information about axonal connections arising from SI. The atlas can be browsed to explore patterns of connectivity and seek answers to specific questions. Bregma levels assigned to the sections provide positional reference. Presence of labeled axons and terminal fields in the images provides evidence of connections between the tracer injection site and a target region. At a more detailed level, it is also possible to analyze the density of the labeling, assumed to reflect the strength of the connections. Further, the topographical distribution of labeled fibers yields additional information on the organization of the connections. In this section, we demonstrate how the atlas system can be used to map and analyze axonal connections (presence, density, and spatial organization) at the level of brain regions, area, and nuclei. Our example analysis will focus on the main ipsilateral subcortical connections of the rat SI cortex based on material from six experimental animals; three with injection sites in SI whisker representations (R602, R604, R606), and three with injection sites in the SI forelimb representation (R601, R603, R605). The position of the injection site centers are given by stereotaxic coordinates, inferred from histological analysis of anatomical landmarks and cytochrome oxidase staining patterns (Fig. 4B–D).

#### Example analysis 1: Presence of labeling

This example serves to illustrate how the Whole Brain Connectivity Atlas can be used to identify SI subcortical projections. SI is known to have connections to a number of subcortical targets (Fig. 1B–C; [31]), including the ipsilateral striatum [32]–[34], thalamus [35], superior colliculus [36], [37], pontine nuclei [37]–[40], and contralateral trigeminal nuclei [41], [42]. To identify these connections in our atlas system with injections in whisker and forelimb representations of SI, we used the bregma levels of individual sections to look up section images centered at each region of interest. The images were inspected in the online viewer tool, and presence of connections confirmed by observation of labeled axons (Fig. 5). For both forelimb and whisker cases, labeled fibers were found in the striatum, thalamus, superior colliculus and pontine nuclei. In two out of three cases with tracer injection in whisker representations, a few labeled fibers were seen in the trigeminal nuclei. Sections were missing at some levels (due to loss during histological processing), thus hampering identification of labeling in the pontine nuclei in one case and in the superior colliculus in another case.

Overall, our findings confirm that the well-known SI subcortical projections are present in our material and can readily be identified in the atlas system.

#### Example analysis 2: Density of labeling

With this example, we aim to show how the Whole Brain Connectivity Atlas can be used to explore not only presence of connections but also differences in the amount of labeling in different SI-subcortical target regions. It is well known that different body parts are represented in SI with different emphasis (or amount of cortical volume; [43]), and that the relative density and spatial extent of subcortical connections varies across different target regions [44]–[46]. Connections from a source region to a given target region can be described by parameters such as densities of axonal plexuses assumed to represent terminal fields of axons and the volume of tissue occupied by the plexuses. Differences in these parameters are readily seen in our atlas, when the amount of labeling is compared across regions in the same cases (Fig. 5). In experiments with tracer injection in SI whisker representations, the most dense axonal labeling is seen in the thalamus. By comparison, the density of labeled axons is clearly lower in the striatum and pontine nuclei, and in the superior colliculus and contralateral trigeminal nuclei, only a few solitary labeled fibers could be observed. In experiments with tracer injection in the SI forelimb representation, the density of labeling in the striatum, thalamus and pontine nuclei appears to be relatively similar. In these cases, a few fibers are observed in the superior colliculus but not in the trigeminal nuclei. While comparisons of amount of labeling across target regions are most valid within the same experiment (since the amount and distribution of labeling is a function of the exact position and size of the injection site, and likely also dependent on other factors specific to a given animal), these observed trends are also consistent across cases. This analysis shows that our system is suited for performing comparisons of the relative amount of connections from one source region to multiple target regions across the brain.

#### Example analysis 3: Topographical distribution of labeling

The orderly arrangement of connections between different parts of the brain is referred to as topographical organization and is considered to reflect important aspects of functional organization [40], [46]–[49]. Characterization of topographically organized distribution patterns is therefore an important aspect of connectivity mapping in the brain. To exemplify how topographical organization can be analyzed with use of the Whole Brain Connectivity Atlas, we identified data in the atlas that could be used to reproduce the well-known topographical arrangement of SI corticothalamic projections. These projections have previously been characterized in detail with use of a dual-tracing approach, demonstrating segregated projections from SI forelimb and whisker representations [50] as well as gradually shifting locations of projections from different SI whisker representations [51].

To reproduce these paradigms with use of the single tracing experiments presented in the atlas, we selected three suitable cases with injection sites located in different SI representations: one with a *Pha*-L injection centered on the SI distal forelimb representation, a second with a BDA injection in the SI whisker D3 representation, and a third with a *Pha*-L injection in the SI whisker D5 representation. Bregma levels for the reticular nucleus, the ventrobasal complex, and the posterior complex of the thalamus were looked up in the stereotaxic atlas of Paxinos and Watson [28] and corresponding images from the three animals (Fig. 6A–C) were superimposed in pairs (Fig. 6D, SI-forelimb and SI-whisker D3; 6F, SI-whisker D3 and SI whisker D5) by aligning the anatomical boundaries to an optimal fit without transforming the images. To facilitate delineation of boundaries between regions, diagrams from Paxinos and Watson [28] taken from corresponding bregma levels were positioned on top of the images and registered by affine transformation, using anatomical landmarks. The resulting composite images clearly show the same topographical distribution patterns as previously demonstrated in dual tracing experiments (Fig. 6E; [50], [51]).

This analysis demonstrates how topographical distribution can be identified using the Whole Brain Connectivity Atlas. The accuracy of the approach is validated by reproducing highly detailed topographical distribution patterns, previously demonstrated by dual tracing experiments.

## Discussion

We present an online data system for mapping and analysis of axonal connectivity in the rat brain, together with a workflow for populating the system with brain-wide series of high resolution images of histological sections containing axonal tracer labeling. We have shown how this system can be used for determining presence of connections, densities, as well as spatial organization of connections throughout the brain. Several methodological choices were made to optimize the experimental tract-tracing approach for whole brain analysis. This included choice of tracer and injection site location and extent.

The employed axonal tracing paradigms are well established, and the tracers BDA and *Pha*-L are both known to have sensitive and specific anterograde labeling properties [15], [16], [52]–[54]. In addition, BDA also gives rise to retrograde labeling, as well as secondary anterograde labeling of collateral fibers (Fig. 1A; [17], [55]–[57]). Thus, some of the BDA labeling in our data sets may not represent direct anterograde connections, in contrast to the *Pha*-L labeling which is known to be purely anterograde [16], [52]. The image repository contains experimental data potentially suitable for elucidation of this phenomenon, but this was considered beyond the scope of the present investigation. More elaborate paradigms with multiple fluorescent tracers were considered disadvantageous since whole-section image acquisition is more challenging with fluorescent labeling. Such paradigms may, however, be relevant with the use of improved slide scanning systems.

The injection sites were targeted to SI, which has some advantages as a model system due to its characteristic and straightforward somatotopic organization [29], [58]–[60]. We here opted for fairly large injection sites, covering the entire cortical depth. The advantage of this choice is that injection sites are easier to standardize, since the large injections are assumed to label connections arising from all layers of cortex. The disadvantage is that the granularity of the results does not allow differentiation of layer-specific connection. We consider this approach suitable for ‘mesoscopic’ levels of investigations, which relate to the organizational features at the level of groups of neurons [20]. To validate the accuracy of the experimental approach, we demonstrated that our single tracer experiments from different animals reproduce well-known topographical patterns, previously demonstrated by dual tracer approaches in single animals [50], [51]. Our results also show that findings are consistent across similar experiments (Fig. 5).

The interpretation, evaluation and reuse of any set of biological experimental data require access to relevant metadata [30], [61]–[65]. Using the Minimum Information about a Neuroscience Investigation suggested by the CARMEN consortium and Gibson and co-workers for experimental electrophysiological studies [30] as a starting point, we have suggested a similar collection of information suitable for axonal tract-tracing experiments. These metadata provide essential information about the experimental animals, experimental parameters, and procedures. Since the spatial distribution and amount of axonal labeling is highly dependent on the position and extent of the tracer injection site, detailed documentation of the injection sites is particularly important. The here presented connectivity atlas provides access to a complete collection of section images, stained to reveal chemo- and cytoarchitecture (cytochrome oxidase and Neutral red staining). In our experiments, tracer injections were positioned on basis of peroperative stereotaxic coordinates, and subsequently related to the SI-characteristic pattern of cytochrome oxidase staining. The accuracy of our anatomical analysis was further improved since stereotaxic positions were assigned to the complete series of sections.

An overall goal in the field is to systematically map the complete brain connectivity of different rodent and primate organisms [9], [20], [66]. In this context, mapping approaches need to be scalable and the ensuing results must be well defined and highly organized. The presented workflow consists of multiple steps, several of which are time-consuming and technically challenging. Many of these steps can be further optimized by use of high-throughput pipelines, utilizing automated equipment (staining instruments and slide scanners) and batch processing, as demonstrated for the Allen Brain Atlas project [67] and other systematic gene expression mapping projects [68], [69]. Experiences accumulated in the current project indicate that workflow bottlenecks in particular relate to technically challenging steps, such as the stereotaxic surgery and application of axonal tracers, determination of the employed section plane, and neuroanatomical evaluation of the injection site locations and section localization parameters. These steps should, nevertheless be possible to standardize across a limited number of collaborating laboratories [20].

We have here demonstrated a feasible workflow for systematic mapping of rodent brain connectivity. The approach can be used to produce connectivity atlases of major projection systems in the brain. An efficient use of such connectivity atlases will require interoperability to other resources in the community, e.g. by use of defined nomenclatures [70], standardized coordinate systems [71], [72], search engine capabilities, and tools for transformation among coordinate systems and atlases (see, e.g. [73], [74]). Logical next steps will be to further optimize analyses, e.g. by 1) introducing automatic filtering of tracer labeling from background staining [75], 2) registering images to a full 3-D atlas framework, and 3) performing automated assignment of location to the labeling [76]. A particular challenge will be to manage generations of data derived through different analyses, and integrate these with the underlying raw data in a searchable database system. Such derived data could, e.g., be filtered or segmented images, qualitative descriptions, or quantitative measurements pertaining to a complete or partial collection of raw image data. For such purposes a backend database is needed in which different data modalities are linked to specific locations in the brain using spatial coordinates and defined anatomical nomenclatures. Further, advanced metadata management will be needed to allow efficient retrieval of various data categories as well as detailed procedures and methods. The technological platform employed in our study [77], [78] can be expanded to accommodate such functionalities.

We conclude that it will be realistic to map complete connections in the rodent brain along the lines proposed by Bohland et al. [20]. Coordinated multi-laboratory or consortium efforts, building on standards at the level of spatial frameworks and data formats, will be required to facilitate such efforts.

## Materials and Methods

Axonal tracing experiments were conducted on four Sprague Dawley and two Wistar adult male rats ranging in weight from 270 g to 350 g (Scanbur BK AS, Nittedal, Norway). All experimental procedures were approved by the institutional animal welfare committee of the University of Oslo (project 05.09) and the Norwegian Animal Research Authority (NARA 08/98201), and in compliance with European Community regulations on animal well being and National Institute of Health guidelines for the care and use of laboratory animals.

### Axonal tracing experiments

Prior to surgery animals were deeply anesthetized by subcutaneous injection (0.2 ml/100 g) of a mixture of equal volumes of Hypnorm® (Janssen Pharmaceutica, Beerse, Belgium) and Dormicum® (5 mg/mL; F. Hoffmann – La Roche, Basel, Switzerland). After 1.5 h 0.1 ml of this mixture was routinely administered s.c. As an exception, one animal (case R603) was anaesthetized by inhalation of 5% isoflurane (Abbott Scandinavia AB, Solna, Sweden) vaporized in a mixture of 30% O_2_ and 70% N_2_. During incision and suturing additional local anesthesia was obtained by application of lidocaine (2%). During surgery, rectal temperature was monitored and maintained within physiological limits with use of a thermostatically controlled heating pad. Post-operatively, analgesia (Temgesic® 0.3 mg/kg s.c. Schering-Plough, Brussels, Belgium) and saline (2 ml 0.9% NaCl, i.p.) was administered. The animal's head was immobilized in a stereotaxic frame (David Kopf Instruments, Tujunga, CA, USA). A small opening was made in the skull and dura overlying the SI, and axonal tracer injections were targeted to individual SI body representations (forelimb or whisker) based on stereotaxic coordinates obtained from previous maps of SI [29], [79], [80].

Each animal received an injection of either biotinylated dextran amine (BDA; Molecular Probes, Eugene, OR, USA) or *Phaseolus vulgaris* leucoagglutinin (*Pha*-L; Molecular Probes, Eugene, OR, USA). To ensure equal distribution of tracer through all cortical layers, several injections were deposited at eight 0.2 mm intervals, starting from a depth of 2.0 mm below the pial surface. BDA (1 µl of 20% solution in buffered saline, pH 7,4) was delivered by pressure injection via glass pipette with a tip diameter of 30 µm attached to the 1 µl Hamilton syringe (Precision Sampling, Baton Rouge, LA, USA). *Pha*-L was delivered iontophoretically through a glass micropipette (tip diameter, 50 µm), using a pulsed current of 10 µA and 7 s on/7 s off cycle, with a total time of 20 min.

After seven days rats were deeply anesthetized with sodium pentobarbital (50 mg/kg i.p.), and transcardially perfused with physiological saline (37°C), followed by 4% paraformaldehyde (37°C, pH 7.4) and 10% sucrose (∼20°C). All the solutions were freshly prepared. The brains were removed immediately and submerged in 30% sucrose for three days. Isolated brains were photographed and the right side of each brain was marked with a shallow cut.

### Histological processing

To ensure a reproducible coronal section plane matching the flat skull orientation employed by stereotaxic atlases [28], each brain was divided into two blocks by orienting a razor blade perpendicular to the brain midline and a horizontal plane touching the most dorsal parts of the olfactory bulb and paraflocculus. The two brain blocks were sectioned 50 µm on a freezing microtome (Microm HM450, Microm Gmbh, Waldorf, Germany) using the cut block face as a basis. Sections were collected in phosphate buffered saline (pH 7.4). Every second section was processed to visualize BDA [53], [81] or *Pha*-L [52]. Briefly, BDA was visualized by incubating sections for 1.5 h in the Vectastain ABC solution (Vector Laboratories, CA, USA) followed by a nickel-enhanced diaminobenzidine (DAB-Ni) reaction. *Pha*-L was visualized by incubating sections overnight with a primary antibody (rabbit anti *Pha*-L, 1∶1000 in 0.05 M TBS-Tx pH 8.0), 90 min with a secondary antibody (goat anti-rabbit, 1∶200 in TBS-Tx), and 1.5 h in the Vectastain ABC solution followed by a nickel-enhanced diaminobenzidine (DAB-Ni) reaction. The sections were mounted on gelatin-coated slides and air-dried overnight. All BDA and *Pha*-L stained sections were counterstained with Neutral red or Thionine, respectively, to reveal cytoarchitectonic boundaries. In addition, alternating sections through the primary somatosensory cortex were stained for cytochrome oxidase using the procedure of Wong-Riley et al. [82]. Sections were mounted, dried and cover-slipped with Eukitt (Kindler, Freiburg, Germany).

### Image acquisition

High-resolution mosaic images of entire sections were obtained through an UPlanApo 20×/0.70 ∞/0.17 (Olympus, Japan) dry objective using a motorized Olympus BX52 microscope running the Virtual Slice module of Neurolucida 7.0 software (MBF Bioscience, Inc., Williston, VT, USA). Adobe Photoshop CS3 (Adobe Systems Inc. San Jose, CA, USA) was used to rotate and center images on a standard sized canvas, and export LZW compressed TIFF images and low-resolution JPEG thumbnail images. TIFF images were further converted to the Zoomify PFF format using the Zoomify Enterprise software (Zoomify Inc., Santa Cruz, CA, USA). To separate the observed labeled fibers from background staining in the images used for the example analysis presented in Figure 5 (panels A′–I′), the following steps were applied: 1) contrast was adjusted using the auto-contrast filter in Adobe Photoshop, 2) hue and saturation were manually adjusted in the blue or red channel to remove Thionine or Neutral red staining, respectively, and 3) images were converted to gray scale mode.

### Assembly of image repository

The images and collected metadata were assembled in an online data repository available through the Rodent Workbench portal (www.rbwb.org). The repository is built on an established technological platform [77], [78]. The resource uses a web interface (http://www.rbwb.org/, choose “Whole Brain Connectivity Atlas”) and provides access to a customized virtual microscopy tool [83] for viewing and navigation within and across section images, based on the Zoomifyer Enterprise application (Zoomify, Inc., Santa Cruz, CA, USA), customized using Flash (Macromedia, Inc., San Francisco, CA, USA). Within the image repository, the images were spatially organized according to serial order and section thickness, and assigned stereotaxic coordinates by anchoring some images to multiple anatomical landmarks (rostral and caudal poles of the cerebral cortex, genu and splenium of the corpus callosum, decussating of the anterior commissure, the oculomotor nerve) and interpolating the remaining image positions. Currently, the data system does not include options for downloading of images, but screen resolution images can be captured using a screen ‘snapshot’ or ‘print’ function. For the example analyses conducted in the present paper we used the original high-resolution TIFF images, but screen snapshots would yield identical results. Image downloading options will be developed for future versions of the system.
